# Association between serum osteocalcin and glucose/lipid metabolism in Chinese Han and Uygur populations with type 2 diabetes mellitus in Xinjiang: two cross-sectional studies

**DOI:** 10.1186/s12944-017-0512-8

**Published:** 2017-07-21

**Authors:** Yuan Chen, Qiang Zhao, Guoli Du, Yancheng Xu

**Affiliations:** 1 Department of Endocrinology, Zhongnan Hospital of Wuhan University, 169 Donghu Road, Wuhan, Hubei 430071 China; 2 Department of Endocrinology, People’s Hospital of Xinjiang Uygur Autonomous Region, Urumqi, China; 3 Department of Cardiology, First Affiliated Hospital of Xinjiang Medical University, Urumqi, China; 4 Xinjiang Key Laboratory of Cardiovascular Disease Research, Urumqi, China; 5 Department of Endocrinology, First Affiliated Hospital of Xinjiang Medical University, Urumqi, China

**Keywords:** Osteocalcin, Glucose/lipid metabolism, Diabetes mellitus, Han, Uygur

## Abstract

**Background:**

Recent studies have shown osteocalcin (OC) plays an important role in regulating glucose and lipid metabolism. Thus, the aim of this study was to investigate the association of OC with glucose and lipid metabolism in patients with type 2 diabetes mellitus (T2DM) in the Chinese Han and Uygur population.

**Methods:**

A total of 1397 T2DM patients (705 Han and 692 Uygur T2DM patients) were enrolled in the present study. Lipid profile, glucose metabolic indices and total OC (TOC) were measured. Homeostasis model assessment of β-cells function (HOMA-β), insulin sensitivity (HOMA-IS) and insulin resistance (HOMA-IR) were also calculated in all participants. Pearson/Spearman correlation analysis and multivariate stepwise regression analysis were adopted to test the relationships between OC and those parameters.

**Results:**

Uygur T2DM patients had significantly higher body mass index (BMI), hemoglobin A1C (HbA1C) and lower TOC compared with their Han counterparts (all *P* < 0.05). HbA1C was negatively associated with TOC in all Uygur and Han T2DM patients (Total: Uygur: *t* = −3.468, *P* = 0.001; Han: *t* = −4.169, *P* < 0.001). BMI was inversely associated with TOC in all Uygur T2DM patients (Males: *t* = −2.893, *P* = 0.014; Females: *t* = −2.250, *P* = 0.027, respectively). Multivariate stepwise regression analysis showed that TOC was positively correlated with HOMA-β in the Uygur male group (β = 2.101, *P* = 0.040) and negatively associated with BMI in all Uygur T2DM patients (Males: β = −1.563, *P* = 0.011; Females: β = −1.284, *P* = 0.016, respectively). No significant differences were observed between TOC and lipid profiles in all participants (all *P >* 0.05).

**Conclusion:**

There were differences in the associations between TOC and glucose metabolism in Han and Uygur T2DM patients, indicating genetic factors may play a role in modulating OC and glucose metabolism in different ethnic population.

## Background

Diabetes mellitus (DM), caused by the interaction of insulin resistance (IR) and insulin deficiency, is a common chronic disease in adults worldwide. The incidence of type 2 DM (T2DM) has been steadily increasing in China over the past few decades. Diabetic osteopathy is a common complication among diabetic patients, characterized by a higher risk of bone fracture, osteoporosis and increased brittleness of bone [1].

Nowadays, bone has been identified as an endocrine organ which plays an important role in the energy metabolism. As an osteoblast-derived hormone, osteocalcin (OC) is an indicator of both osteoblast activity and bone formation. Both carboxylated and uncarboxylated forms of OC are found in serum, with the undercarboxylated OC (ucOC) being the main component of total OC (TOC). Recently, increasing attention has been directed toward the role of OC in modulating glucose and lipid metabolism. Lee et al. showed that OC is a bone messenger affecting both adipocytes and insulin-producing β-cells. It increases β-cells proliferation, insulin secretion, insulin sensitivity, adiponectin expression and energy expenditure by upregulating the expression of adiponectin gene in adipocytes [2]. Adiponectin and its receptors are expressed in human osteoblasts. Adiponectin stimulates the proliferation, differentiation and mineralization of osteoblastic cells [3, 4]. As one of adipocytokines, adiponectin also enhances insulin sensitivity, indicating there is an interaction among bone, adipose-derived factors and glucose/lipid metabolism.

Several clinical studies have investigated the association between OC and glucose/lipid metabolism [5–8]. However, the results were inconsistent, probably owing to the different ethnic origin which is specific to the study population enrolled in the different studies. There were few studies investigating the differential patterns for regulating glucose and lipid metabolism by OC in Chinese Han and Uygur T2DM patients, so we perform this analysis to investigate the association of serum TOC with glucose and lipid metabolism in Chinese Han and Uygur T2DM patients.

## Methods

### Ethical approval of the study protocol

This study protocol was approved by the Ethics Committee of the People’s Hospital of Xinjiang Uygur Autonomous Region. All participants provided written informed consent to participate the research.

### Study design and population

Two independent cross-sectional studies conducted at the Xinjiang Uygur Autonomous Region were included. A total of 1397 Han and Uygur T2DM patients admitted to the endocrine department of the People’s Hospital of Xinjiang Uygur Autonomous Region between January 2014 and October 2015 were recruited. Individuals were excluded from this study if they have: acute or severe chronic DM complications, current cancer, chronic/acute kidney or liver disease, bone-altering conditions (hyperparathyroidism, bilateral hip surgery), oral medications known to influence bone and calcium metabolism, such as vitamin D, bisphosphonates and estrogen.

### Definition of DM and its risk factors

DM was defined as history of DM or was taking anti-diabetic medications or had fasting/non-fasting glucose ≥7.0 mmol/L/≥ 11.1 mmol/L. Body mass index (BMI) was calculated by dividing the weight in kilograms by the height in meters squared. Waist circumference (WC) was measured at midpoint between the last palpable rib and the suprailiac crest using a measuring tape parallel to the floor with the subjects standing and breathing normally. Obesity was defined as BMI ≥ 28.0 kg/m^2^ [9].

### Anthropometric measurements

Fasting blood samples were collected from all participants for the assessment of biochemical indices. Fasting plasma glucose (FPG), glycated hemoglobin A1C (HbA1C), total cholesterol (TC), low density lipoprotein-cholesterol (LDL-C), high density lipoprotein-cholesterol (HDL-C), triglycerides (TG), calcium, phosphorus, alkaline phosphatase (ALP), parathyroid hormone (PTH), 25-hydroxyvitamin D3 [25(OH)VD3], procollagen type I N-terminal propeptide (P1NP) and β-C-terminal telopeptide of type I collagen (β-CTX) were measured using standard enzymatic methods in the central laboratory of the People’s Hospital of Xinjiang Uygur Autonomous Region. Fasting plasma insulin (FINS)/C-peptide (FCP), 2 h–post OGTT plasma glucose (2 h–PG) and C-peptide (2 h–CP) were measured using enzyme-linked immunosorbent assay method. The assessment of insulin sensitivity was estimated by homeostasis model assessment-IR (HOMA-IR) based on FPG and insulin measurements as follows: HOMA-IR = [FINS (mU/L) × FPG (mmol/L)]/22.5 [6]. Homoeostasis model assessment for insulin secretion (HOMA-IS) was calculated using the formulas previously described [10]. To estimate β-cells function, HOMA-β was calculated as follows: (20 × FINS)/(FPG-3.5). Serum TOC was measured using the electrochemical luminescence method. Bone mass density (BMD) was measured using dual-energy x-ray absorptiometry (LINK Osteocore 3, France) with intra- and inter-assay coefficient of variation below 1%. BMD at the hip, anteroposterior lumbar spine (L1–4), greater trochanter (GT) and femoral neck were measured (grams per square centimeter).

### Statistical methods

All statistical analyses were performed with the SPSS for Windows (version 17.0, SPSS Inc., Chicago, IL, USA). Continuous data were examined for normality and expressed as mean ± standard deviation (SD) or median and compared with the Student’s t test or Wilcoxon test as appropriate. Categorical variables were summarized as percentages and compared with the Chi-square test. Pearson/Spearman analysis model was used to assess the association of TOC with anthropometric variables, glucose and lipid metabolic indices. A multivariate stepwise analysis was performed to test for the association between TOC and other independent variables after adjustment for confounding variables. A 2-sided *P* < 0.05 was considered to indicate statistical significance.

## Results

During the study period, a total of 705 Han T2DM patients (59.12 ± 8.77 years, 47.98% men) and 692 Uygur T2DM patients (59.95 ± 7.38 years, 49.50% men) were enrolled in the present study.

### Baseline characteristics of the study population stratified by ethnicity and sex

Clinical characteristics of all participants stratified by ethnicity and sex were summarized in Table 1. There were no differences between Han and Uygur T2DM patients with respect to age, course of disease, systolic blood pressure (SBP), diastolic blood pressure (DBP), BMD-neck, BMD-GT, BMD (L1–4), previous medications and prevalence of comorbidities. For total, males and females, Uygur T2DM patients had higher WC, BMI and higher prevalence of obesity compared with their Han counterparts (all *P* < 0.05). In the female group, Uygur females had significantly higher BMD-neck values than Han females (0.73 ± 0.11 g/cm^2^ vs. 0.70 ± 0.14 g/cm^2^, *P* = 0.024).

**Table 1:** Baseline characteristics of study population stratified by ethnicity and sex

Variables	Total			Male			Female		
	Uygur (*n* = 692)	Han (*n* = 705)	*P* value	Uygur (*n* = 332)	Han (*n* = 349)	*P* value	Uygur (*n* = 360)	Han (*n* = 356)	*P* value
Age, years	59.95 ± 7.38	59.12 ± 8.77	0.480	58.88 ± 7.29	61.70 ± 8.75	0.132	60.82 ± 7.43	58.92 ± 9.54	0.167
Duration of T2DM, years	8.71 ± 5.87	8.87 ± 7.14	0.658	8.36 ± 5.77	8.46 ± 6.85	0.617	9.00 ± 7.76	9.4 ± 5.93	0.462
WC, cm	106.60 ± 12.87	94.59 ± 11.55	<0.001	104.37 ± 12.06	96.69 ± 9.56	0.003	102.88 ± 13.48	93.22 ± 9.56	<0.001
SBP, mmHg	128.97 ± 22.37	132.21 ± 19.61	0.205	126.09 ± 20.36	131.74 ± 19.90	0.217	131.32 ± 19.90	132.80 ± 19.25	0.394
DBP, mmHg	79.28 ± 10.96	78.94 ± 11.55	0.581	79.91 ± 9.67	80.50 ± 11.26	0.608	78.72 ± 11.90	76.96 ± 11.12	0.053
BMI, kg/m^2^	27.89 ± 5.18	25.61 ± 3.61	<0.001	28.17 ± 18.06	25.59 ± 3.22	0.010	28.37 ± 6.49	25.63 ± 4.04	<0.001
BMD-neck, g/cm^2^	0.76 ± 0.12	0.74 ± 0.15	0.211	0.80 ± 0.12	0.79 ± 0.15	0.136	0.73 ± 0.11	0.70 ± 0.14	0.024
BMD-GT, g/cm^2^	0.73 ± 0.14	0.74 ± 0.15	0.251	0.79 ± 0.12	0.79 ± 0.13	0.165	0.67 ± 0.13	0.67 ± 0.14	0.993
BMD-hip, g/cm^2^	0.97 ± 0.82	0.91 ± 0.15	0.161	1.09 ± 1.2	0.95 ± 0.14	0.165	0.87 ± 0.14	0.85 ± 0.16	0.237
BMD(L1–4), g/cm^2^	0.88 ± 0.16	0.90 ± 0.17	0.261	0.94 ± 0.15	0.96 ± 0.17	0.135	0.82 ± 0.18	0.84 ± 0.16	0.999
Comorbidities									
Obesity, *n* (%)	276 (39.88)	148 (20.99)	<0.001	118 (35.54)	72 (20.63)	<0.001	168 (46.67)	94 (26.39)	<0.001
Diabetic nephropathy, *n* (%)	128 (18.50)	118 (16.74)	0.388	65 (19.58)	58 (16.62)	0.316	63 (17.50)	60 (16.85%)	0.819
Diabetic retinopathy, *n* (%)	140 (20.23)	133 (18.87)	0.520	72 (21.69)	69 (19.77)	0.537	68 (18.89)	64 (17.98)	0.753
Peripheral neuropathy, *n* (%) neuropathy	228 (32.95)	201 (28.51)	0.072	119 (35.84)	103 (29.51)	0.078	109 (30.28)	98 (27.53)	0.084
Coronary heart disease, *n* (%)	158 (22.83)	141 (20.00)	0.086	81 (24.40)	72 (20.63)	0.239	77 (21.39)	69 (19.38)	0.505
Osteoporosis, *n* (%)	129 (18.64)	150 (21.28)	0.218	39 (11.75)	42 (12.03)	0.628	90 (25.00)	108 (30.34)	0.110
Previous medications									
Sulfonylureas, *n* (%)	184 (26.59)	158 (22.41)	0.069	96 (28.92)	82 (23.50)	0.108	88 (24.44)	76 (21.35)	0.324
Metformin, *n* (%)	485 (70.09)	502 (71.21)	0.646	236 (71.08)	239 (68.48)	0.460	249 (69.17)	263 (73.88)	0.163
Glucosidase inhibitor, *n* (%)	363 (52.46)	378 (53.62)	0.664	179 (53.92)	187 (53.58)	0.930	184 (51.11)	191 (53.65)	0.496
Insulin, *n* (%)	110 (15.90)	99 (14.04)	0.271	57 (17.17)	48 (13.75)	0.271	53 (14.72)	51 (14.33)	0.880
Anti-dyslipidemic, *n* (%)	80 (11.56)	88 (12.48)	0.878	41 (12.35)	49 (14.04)	0.515	39 (10.83)	46 (12.92)	0.388

### Laboratory characteristics of the study population

Table 2 showed the laboratory findings of the study stratified by ethnicity and sex. There were no differences with respect to the PTH, calcium, FCP, FINS, HDL-C, PINP, HOMA-IR, HOMA-IS and HOMA-β between Han and Uygur T2DM patients (all *P* > 0.05). Han T2DM patients had significantly higher TOC compared with Uygur participants (Total: 19.30 ± 12.53 ng/mL vs. 13.91 ± 7.45 ng/mL, *P* < 0.001; Males: 19.10 ± 11.71 ng/mL vs. 12.69 ± 7.22 ng/mL, *P* < 0.001; Females: 19.60 ± 12.33 ng/mL vs. 15.42 ± 7.46 ng/mL, *P* < 0.001). For total participants, males and females, Uygur T2DM patients had significantly higher FPG, 2 h–PG, high sensitivity C-reactive protein (hs-CRP) and HbA1C than their Han counterparts (all *P* < 0.05). In the total and female groups, Uygur T2DM patients had higher ALP, LDL-C, TC, TG and β-CTX and lower 25(OH)VD3, creatinine and 2 h–CP (all *P* < 0.05).

**Table 2:** Laboratory parameters of study population

Variables	Total			Males			Females		
	Uygur	Han	*P* value	Uygur	Han	*P* value	Uygur	Han	*P* value
	(*n* = 692)	(*n* = 705)		(*n* = 332)	(*n* = 349)		(*n* = 360)	(*n* = 356)	
TOC, ng/mL	13.91 ± 7.45	19.30 ± 12.53	<0.001	12.69 ± 7.22	19.10 ± 11.71	<0.001	15.42 ± 7.46	19.60 ± 12.33	<0.001
25(OH) VD3, ng/mL	6.74 (3.62–11.23)	11.47 (6.87–18.57)	<0.001	10.61 ± 7.73	14.16 ± 9.44	0.183	7.80 ± 6.72	12.68 ± 10.21	<0.001
PTH, pg/mL	34.40 (24.15–49.30)	34.10 (23.28–48.57)	0.890	32.75 ± 19.41	34.87 ± 20.21	0.567	31.03 ± 29.87	34.66 ± 20.22	0.489
Calcium, mmol/L	2.26 ± 0.14	2.28 ± 0.17	0.927	2.16 ± 0.14	2.26 ± 0.12	0.924	2.23 ± 0.15	2.30 ± 0.12	0.875
Phosphorus, mmol/L	1.14 ± 0.21	1.11 ± 0.22	0.567	1.16 ± 0.22	1.08 ± 1.23	0.395	1.15 ± 1.29	1.14 ± 0.20	0.780
ALP, U/L	88.31 ± 27.22	70.75 ± 21.21	<0.001	81.16 ± 23.78	67.37 ± 19.16	0.242	94.44 ± 28.52	74.98 ± 22.91	<0.001
hs-CRP, mg/L	3.35 (1.63–6.45)	1.62 (0.86–3.78)	<0.001	2.73 (1.30–5.20)	1.60 (0.74–1.32)	0.001	4.24 (2.17–7.78)	1.46 (0.83 ~ 3.70)	<0.001
Creatinine, μmol/L	62.25 ± 21.62	71.17 ± 23.56	<0.001	75.17 ± 22.22	77.59 ± 22.46	0.596	56.98 ± 17.23	62.92 ± 22.39	<0.001
FPG, mmol/L	9.58 ± 3.70	8.18 ± 3.39	<0.001	9.22 ± 3.34	8.30 ± 3.45	0.013	9.87 ± 3.95	8.02 ± 3.31	<0.001
2 h–PG, mmol/L	18.23 ± 5.81	16.86 ± 6.52	<0.001	22.80 ± 9.50	17.49 ± 5.90	0.006	18.83 ± 5.67	16.33 ± 6.64	<0.001
FINS, mU/L	9.05 (5.30–14.77)	7.49 (5.02–11.4)	0.230	8.17 ± 7.63	7.81 ± 7.29	0.115	4.13 ± 3.44	5.12 ± 3.85	0.424
FCP, ng/mL	2.33 ± 1.13	2.34 ± 1.26	0.217	1.86 ± 1.32	1.79 ± 1.54	0.480	1.85 ± 1.39	1.88 ± 1.54	0.394
2 h–CP, ng/mL	5.14 ± 2.84	5.86 ± 3.20	<0.001	4.22 ± 3.03	4.43 ± 3.52	1.620	4.13 ± 3.44	5.12 ± 3.85	0.001
HbA1C, %	9.56 ± 2.11	8.49 ± 2.07	<0.001	9.48 ± 2.14	8.60 ± 2.14	0.021	9.62 ± 2.07	8.51 ± 3.71	<0.001
TC, mmol/L	4.84 ± 1.34	4.47 ± 1.15	<0.001	4.76 ± 1.43	4.36 ± 1.21	0.578	4.89 ± 1.25	4.60 ± 1.06	0.002
TG, mmol/L	2.24 ± 1.77	2.02 ± 1.68	0.018	1.93 (1.38–3.25)	1.69 (1.38–2.25)	0.187	1.80 (1.32–3.35)	1.60 (1.21–2.27)	0.031
LDL-C, mmol/L	2.90 ± 1.00	2.60 ± 0.88	<0.001	2.80 ± 0.99	2.54 ± 0.90	0.759	2.99 ± 1.00	2.67 ± 0.85	<0.001
HDL-C, mmol/L	1.65 ± 0.78	1.69 ± 1.14	0.334	1.62 ± 0.96	1.66 ± 1.05	0.334	1.67 ± 0.59	1.72 ± 1.23	0.357
P1NP, ng/mL	49.29 ± 28.33	46.24 ± 21.96	0.345	43.35 ± 13.36	47.87 ± 21.57	0.750	54.00 ± 33.07	41.87 ± 21.57	0.275
β-CTX, ng/mL	0.46 ± 0.27	0.41 ± 0.20	0.043	0.41 ± 0.21	0.38 ± 0.20	0.081	0.50 ± 0.31	0.40 ± 0.21	0.012
HOMA-IR	3.72 (2.07–5.07)	2.48 (0.15–4.17)	0.410	2.71 (1.16–4.92)	2.02 (1.38–4.20)	0.205	3.52 (1.60–5.66)	2.67 (1.60–3.90)	0.501
HOMA-IS	0.37 (0.18–0.48)	0.41 (0.24–0.69)	0.137	0.37 (0.20–0.86)	0.49 (0.24–0.73)	0.126	0.28 (0.18–0.63)	0.28 (0.26–0.63)	0.083
HOMA-β, %	39.59 (17.33–78.82)	45.41 (21.98–87.72)	0.561	29.74 (10.21–59.62)	38.30 (19.11–72.48)	0.213	38.42 (14.16–79.91)	54.23 (31.14–101.32)	0.337

### Pearson/Spearman correlation analysis

The correlations between TOC and anthropometric variables, glucose and lipid metabolic parameters were presented in Tables 3. BMI was inversely correlated with TOC in all Uygur T2DM patients (Total: *r* = −0.118, *P* = 0.003; Males: *r* = −0.168, *P* = 0.025; Females: *r* = −0.164, *P* = 0.030), while it was only negatively associated with TOC in the Han total and Han female group (Total: *r* = −0.119, *P* = 0.004; Females: *r* = −0.262, *P* < 0.001). WC was demonstrated to be inversely associated with TOC only in the Han female group (*r* = −0.181, *P* = 0.004). HbA1C was negatively associated with TOC in all T2DM patients (Total Uygur: *r* = −0.143, *P* < 0.001; total Han: *r* = −0.181, *P* < 0.001; Uygur males: *r* = −0.171, *P* = 0.002; Han males: *r* = −0.149, *P* = 0.008; Uygur females: *r* = −0.215, *P* < 0.001; Han female: *r* = −0.207, *P* < 0.001). 2 h–PG was inversely related with TOC in all Han and Uygur female participants, while FPG was inversely associated with TOC in the Han and Uygur female group (all *P* < 0.05). When it comes to P1NP, PTH and phosphorus, they were positively associated with TOC in all Han and Uygur T2DM patients (all *P <* 0.05). There was no significant correlation between serum TOC and lipid metabolic parameters, including TC, LDL-C and TG in all Han and Uygur T2DM patients.

**Table 3:** Spearman/Pearson analysis between TOC and glucose/lipid metabolic indices

Variables	Total				Males				Females			
	Uygur		Han		Uygur		Han		Uygur		Han	
	r	*P*	r	*P*	r	*P*	r	*P*	r	*P*	r	*P*
Age	−0.047	0.259	−0.024	0.537	0.200	0.733	0.210	0.712	69.000	0.184	0.110	0.860
Duration of T2DM	0.038	0.329	0.047	0.259	0.134	0.210	−0.040	0.480	0.050	0.340	0.134	0.310
BMI	−0.118	0.003	−0.119	0.004	−0.168	0.025	−0.049	0.390	−0.164	0.030	−0.262	<0.001
WC	−0.026	0.514	−0.128	0.002	−0.039	0.503	−0.012	0.829	−0.080	0.132	−0.181	0.004
25(OH) VD3	0.025	0.435	−0.019	0.665	0.019	0.763	−0.012	0.838	0.052	0.347	0.001	0.987
PTH	0.162	<0.001	0.220	<0.001	0.135	0.031	0.263	<0.001	0.180	0.001	0.187	0.004
Calcium	−0.002	0.965	−0.003	0.946	−0.073	0.291	0.086	0.182	0.057	0.350	0.022	0.757
Phosphorus	0.217	<0.001	0.167	<0.001	0.204	0.003	0.500	0.444	0.228	<0.001	0.273	<0.001
ALP	0.042	0.538	0.043	0.470	−0.020	0.807	0.032	0.699	0.099	0.185	−0.024	0.785
FPG	−0.115	0.003	−0.125	0.003	−0.078	0.179	−0.080	0.168	−0.152	0.004	−0.165	0.009
2 h–PG	−0.132	0.002	−0.127	<0.001	−0.105	0.092	−0.178	0.030	−0.166	0.003	−0.159	0.016
FINS	0.064	0.254	0.084	0.077	0.002	0.983	0.111	0.185	0.096	<0.001	0.035	0.699
FCP	0.009	0.831	0.069	0.134	0.164	0.009	0.192	0.002	0.183	0.002	0.076	0.263
2 h–CP	0.057	0.192	0.077	0.095	−0.068	0.289	0.088	0.163	0.168	0.005	0.037	0.594
HbA1C	−0.143	<0.001	−0.181	<0.001	−0.171	0.002	−0.149	0.008	−0.215	<0.001	−0.207	<0.001
TC	0.004	0.929	0.013	0.764	0.031	0.603	−0.025	0.685	−0.027	0.616	0.016	0.805
TG	−0.035	0.382	−0.032	0.454	−0.148	0.306	−0.077	0.188	−0.005	0.933	−0.000	0.999
LDL-C	−0.051	0.206	−0.007	0.864	−0.072	0.228	−0.006	0.919	−0.030	0.582	−0.025	0.706
HDL-C	0.176	0.360	0.091	0.154	0.153	0.207	0.065	0.174	0.004	0.842	0.001	0.875
P1NP	0.390	<0.001	0.528	<0.001	0.305	<0.001	0.510	<0.001	0.452	<0.001	0.506	<0.001
β-CTX	0.405	<0.001	0.522	<0.001	0.335	<0.001	0.543	<0.001	0.456	<0.001	0.472	<0.001
HOMA-IR	0.094	0.015	0.072	0.083	0.148	0.010	0.018	0.754	0.040	0.445	0.113	0.224
HOMA-IS	0.033	<0.001	0.023	0.724	2.690	0.001	0.380	0.668	0.228	0.005	0.116	0.068
HOMA-β	0.130	0.035	0.140	0.002	0.170	0.025	0.082	0.144	0.014	0.785	0.157	0.011

### Multivariate stepwise regression analysis

Multivariate stepwise regression analysis of the associations between TOC and multiple parameters stratified by ethnicity and sex were presented in Table 4. In a multivariate stepwise regression model with TOC as the dependent variable, we identified HbA1C was negatively correlated with TOC in all Han and Uygur participants. BMI was inversely associated with TOC in all Uygur participants, including males and females. While HOMA-β was only positively associated with TOC in the Uygur male group. In addition, β-CTX was positively associated with TOC in all Han and Uygur participants.

**Table 4:** Multivariable association of TOC with other independent selected variables

Groups	Variables	β	Sd. E	Standardized β	t	*P* value
Total Uygur group	HbA1C	−0.663	0.191	−0.180	−3.468	0.001
	P1NP	0.100	0.023	0.296	4.337	<0.001
	ß-CTX	11.096	2.289	0.331	4.847	<0.001
	BMI	−0.184	0.072	−0.097	−2.567	0.011
Total Han group	HbA1C	−0.536	0.129	−0.157	−4.169	<0.001
	P1NP	0.097	0.015	0.313	6.363	<0.001
	β-CTX	10.249	1.618	0.316	6.335	<0.001
Uygur Males group	HbA1C	−0.691	0.202	−0.247	−3.979	0.002
	β-CTX	0.223	0.079	0.341	2.836	0.001
	BMI	−0.454	0.167	−0.171	−2.893	0.014
	FCP	0.639	0.292	0.151	2.185	0.031
	HOMA-β	0.016	0.048	0.207	3.567	0.001
Han Males group	HbA1C	−0.451	0.164	0.189	−2.185	0.031
	P1NP	0.088	0.017	0.425	5.230	<0.001
	β-CTX	6.890	1.887	0.292	3.655	<0.001
Uygur Females group	HbA1C	−0.613	0.172	−0.164	−3.564	0.003
	P1NP	0.070	0.028	0.278	2.499	0.015
	β-CTX	11.036	3.285	0.398	3.359	0.001
	BMI	−0.320	0.142	−0.158	−2.250	0.027
Han Females group	HbA1C	−0.608	0.200	−0.186	−3.036	0.003
	P1NP	0.071	0.024	0.240	2.994	0.003
	β-CTX	13.721	2.469	0.445	4.749	<0.001

To investigate the association between independent risk factors of HbA1C, BMI and HOMA-β, a multivariate regression analysis was performed with HbA1C as the dependent variable and age, BMI, TOC, FPG, FINS, FCP, TG, TC, HDL-C and LDL-C as independent variables. We identified a significantly negative association between TOC and HbA1C in all participants (Han: β = −0.045, *P* = 0.015; Uygur:β = −0.034, *P* = 0.014) (Table 5). While in a multivariate stepwise regression analysis performed with BMI as the dependent variable, we found TOC was negatively associated with BMI only in the Uygur groups (Males: β = −1.563, *P* = 0.011; Females: β = −1.284, *P* = 0.016) (Table 6). Furthermore, TOC was positively associated with HOMA-β in the Uygur male group after adjusting for confounding variants (β = 2.101, *P* = 0.040) (Table 6).

**Table 5:** Multivariate stepwise regression analysis of HbA1C and other parameters

	Uygur						Han					
Variables	HbAC1		P1NP		β-CTX		HbA1C		P1NP		β-CTX	
	β	*P* value	β	*P* value	β	*P* value	β	*P* value	β	*P* value	β	*P* value
TOC	−0.034	0.014	0.443	<0.001	0.005	<0.001	−0.045	0.015	1.933	<0.001	0.015	<0.001
FPG	0.280	<0.001	-	-	-	-	0.313	<0.001	-	-	-	-
2 h–PG	0.098	<0.001	-	-	-	-	0.070	0.001	-	-	-	-
ALP	-	-	0.213	<0.001	0.002	<0.001	-	-	0.190	0.001	0.002	<0.001
PTH	-	-	0.152	0.003	0.001	0.041	-	-	-	-	-	-
Calcium	-	-	-	-	−0.206	0.033	-	-	-	-	−0.555	<0.001

**Table 6:** Multivariate stepwise regression analysis of HOMA-β and other parameters among Uygur population

Variables	Uygur males				Uygur females	
	BMI		HOMA-β		BMI	
	β	*P* value	β	*P* value	β	*P* value
TOC	−1.563	0.011	2.101	0.040	−1.284	0.016
FPG	-	-	−5.476	0.001	−5.933	<0.001
FCP	0.906	0.016	-	-	13.965	0.005
FINS	0.134	0.008	1.232	<0.001	-	-

## Discussion

It is well known that T2DM is a complex metabolic disease caused by the interaction of genetic, environmental and other risk factors. Earlier clinical studies reported serum OC was associated with glucose tolerance in middle-aged men [5] and T2DM patients [6]. In the present study, serum concentrations of TOC, HbA1C and BMI were statistically different between Han and Uygur T2DM patients. By multivariate analysis, the concentration of TOC was inversely associated with HbA1C for Han and Uygur T2DM participants and it was also inversely correlated with BMI for Uygur T2DM participants. In addition, it was positively associated with HOMA-β for Uygur male participants, indicating there is an interaction between TOC and glucose metabolism. The results indicated that genetic factors may play a role in modulating TOC and glucose metabolism in different ethnic population.

Emerging evidences from clinical and animal studies have shown that bone interacts with glucose metabolism by regulating insulin secretion from pancreas as well as adipokines from adipose tissue. As a bone-derived hormone produced and secreted specifically by osteoblasts, OC has been widely used as a biochemical marker of bone formation and bone turnover [11]. Recent studies in mice demonstrated the potential effect of skeleton in glucose and lipid homeostasis, which was regulated by OC,indicating OC may be involved in the regulation of glucose and lipid metabolism in human.

The association between serum OC and energy metabolism has been extensively studied in T2DM patients. However, so far contrasting results have been reported. Iki et al. investigated the association of the serum OC and glycemic status and IR in an elderly Japanese male population and demonstrated that serum OC was negatively correlated with FPG and HbA1C [12]. Kanazawa et al. surveyed 101 postmenopausal women and 152 men diagnosed with T2DM and concluded serum OC was negatively correlated with FPG and HbA1C [13]. However, Wang et al. reported there was no relationship between OC and FPG [14]. In our study, TOC and glucose metabolism has been analyzed in 705 Han T2DM patients and 692 Uygur T2DM patients. We found Uygur participants had higher BMI and lower serum TOC than their Han participants. Our result is consistent with Cardiovascular Risk Survey (CRS) study which was performed from June 2007 to March 2010 [15], demonstrating Uygur population had a significantly higher prevalence of obesity and DM than Han population. Life styles, living habits, living environments and genetic factors, especially the genes involved in obesity may contribute to ethnic variance in BMI in the present study. Previous studies have reported obesity contributes to the pathogenesis of T2DM mainly by promoting IR [16, 17]. The mechanisms whereby obesity predisposes to IR are incompletely understood. Central fat accumulation, intraorgan lipid deposition, plasma free fatty acid accumulation, inflammation and adipocytokine release may contribute to the IR from the adiposity perspective [18–21].

In a previous study conducted in the black, Hispanic and white population enrolled in the Boston Area Community Health/Bone (BACH/Bone) Survey by Travison et al. [22], they found there are racial/ethnic heterogeneity in measures of bone mass and density including OC and concluded that such ethnic heterogeneity may be explained by the variation in body composition, diet and sociodemographic factors. We also found ethnic variation in OC concentrations between Han and Uygur T2DM patients, which confirmed the results of previous study. Our study demonstrated that TOC was negatively correlated with HbA1C for all Han and Uygur T2DM patients. The inverse association between TOC and HbA1C was retained after adjustment of major risk factors, indicating TOC may modulate glucose metabolism through pathways beyond established risk factors.

OC has been reported to regulate glucose metabolism by increasing insulin secretion and improving glucose intolerance. Prior animal studies showed that mice lacking OC developed hyperglycemia and glucose intolerance due to IR [2]. The administration of OC in vitro and in wild-type mice could improve glucose homoeostasis by enhancing the expression of insulin genes and genes necessary for β-cell proliferation [2, 23]. Kover et al. confirmed OC can protect β-cells from the negative effects of glucose-induced oxidative stress by reducing TXNIP expression, thereby preserving β-cell function and survival in rat model [24].

A few studies have investigated the relationship between OC and glucose metabolism in the general population. In a large cross-sectional analysis of older men aged 70 to 89 years residing in the community of Perth, Western Australia, higher ucOC was found inversely associated with reduced diabetic risk [25]. Shu et al. found OC at baseline was negatively related to the risk of incident T2DM during a 3-year follow-up [26]. In addition, maternal oral administration of ucOC during gestation protected high-fat, high-sucrose diet-fed female offspring from metabolic disorders induced by maternal obesity [27]. All these studies support a role for OC as a modulator of glucose metabolism, indicating the mechanistic link between OC and glucose metabolism.

The association between serum OC and BMI has been comprehensive studied in obesity animal model [28]. Only some clinical studies investigated the association of OC and BMI [6, 29, 30]. Hu et al. investigated the association of serum TOC and plasma glucose and BMI in a total of 2032 healthy Chinese women in Shanghai [31], and demonstrated that serum TOC was negatively correlated with BMI. Chin KY et al. surveyed serum TOC and indices of obesity and lipid parameters in a total of 373 aging men from the Malaysian, and indicated that low serum TOC level was more likely to be associated with high BMI [32]. To the best of our knowledge, it was the first study to examine the association between TOC and BMI in Chinese Uygur population. In the present study, we showed Uygur T2DM patients had higher BMI than their Han counterparts, and TOC was negatively associated with BMI for all Uygur T2DM patients, which was consistent with previous studies. However, such relationship has been found only in Han female participants. A possible reason for this discrepancy between ethnicity may be related to the higher prevalence of obesity and unhealthy life styles that were specific to Uygur population. Until now, the correlation between serum OC and BMI is not completely understood. Further studies are needed to confirm the relationship between OC and BMI.

Prior animal studies showed that OC induces insulin release in β-cells, improves insulin sensitivity and directly regulates metabolic phenotype [2, 23]. OC knock-out mice showed a profoundly deranged metabolic phenotype including IR, glucose intolerance and abnormal increased visceral fat [23]. While given OC, models of obese mice gained significantly less weight and smaller fat pads [23]. Ma et al. reported the serum OC was positively correlated with HOMA-β in 495 T2DM males [33]. Kunutsor et al. conducted a systematic review and proved serum OC level was positively associated with HOMA-β [34]. Our results confirmed these studies. In the present study, we showed that TOC was positively correlated with HOMA-β for Uygur male participants. Our result supported the hypothesis that OC could enhance β-cells function. Such relationship has not been found in Han and Uygur female participants, which may be related to the limited number of female T2DM patients and the hypothesis that serum oestrogen may disturb the metabolism of OC. In Han male participants, we found no relationship between TOC and HOMA-β. Different ethnic origin and genetic background may be helpful to explain such ethnic variance.

Dyslipidemia, commonly present in patients with DM, is a well-known atherosclerotic cardiovascular disease (ASCVD) risk factors. For patients with ASCVD or with a moderately increased 10-year risk of ASCVD events, aggressive LDL-lowering is the main pharmacological treatment model. However, some patients with CAD still failed to achieve the target level of lipid levels despite taking aggressive LDL-lowering medicine. Nowadays, nutraceuticals and functional food ingredients have been recommended as adjuvants in reducing overall cardiovascular risk induced by dyslipidemia [35]. OC have been reported to play an important role in energy metabolism. While it remained controversial as to whether there was significant association between serum OC and lipid metabolism. Zhou et al. surveyed the association of serum OC and lipid parameters in Chinese subjects, and concluded that HDL-C and TG level were inversely associated with serum OC in Chinese males [36]. Kanazawa et al. reported serum OC was negatively associated with triglyceride in a longitudinal study conducted in Japan T2DM patients [37]. While Bao et al. found no relationship between serum OC levels and lipid metabolism in subjects with normal glucose tolerance and normal BMI in Chinese men [30]. Brownbill et al. also found no relationship between OC level and serum TG [38]. Our study also failed to find the association between serum OC and lipid profiles in Chinese Han and Uygur T2DM patients, which may be due to the difference in diet specific to ethnic population and the selection standard or criteria for the subjects in different studies.

More than 7 chromosomal loci associated with T2DM have been identified to date in the populations of European by genome-wide association (GWA) studies [39–43]. Considering the distinct ethnic origin and genetic background between Han and Uygur population, and the important role played by OC in regulating glucose metabolism, further studies are needed to study the role of polymorphism of OC genes in modulating susceptibility to DM in Chinese Han and Uygur population.

This study should be viewed in light of several limitations. First, the lack of a healthy control group is the main limitation of our study. Second, this study was a single-center experience and enrolled only Chinese Han and Uygur population. Whether our results can be applied to other races remains to be determined. Third, we were unable to determine the impact of lifestyle on the association between OC and metabolic parameters. Finally, we did not measure ucOC and assess the direct effect of ucOC on glucose metabolism.

## Conclusion

In summary, our study demonstrated that there was significantly different association between TOC and glucose metabolism and β-cells function in Han and Uygur T2DM patients, indicating genetic factors may play a role in modulating TOC and glucose metabolism. Further studies are needed to explore the impact of TOC on glucose metabolism in different ethnic humans.

## Abbreviations

25(OH)VD3: 25-hydroxyvitamin D3
2 h–PG: 2 h–post OGTT plasma glucose
ALP: Alkaline phosphatase
BMD: Bone mass density
BMI: Body mass index
DBP: Diastolic blood pressure
FCP: Fasting plasma C-peptide
FINS: Fasting plasma insulin
FPG: Fasting plasma glucose
HbA1C: Glycated hemoglobin
HDL-C: High density lipoprotein-cholesterol
HOMA-IR: Homeostasis model assessment of insulin resistance
HOMA-IS: Homeostasis model assessment of insulin sensitivity
HOMA-β: Homeostasis model assessment of β-cell function
LDL-C: Low density lipoprotein-cholesterol
P1NP: Procollagen type I N-terminal propeptide
PTH: Parathyroid hormone
SBP: Systolic blood pressure
SD: Standard deviation
T2DM: Type 2 diabetes mellitus
TC: Total cholesterol
TG: Triglyceride
TOC: Serum total osteocalcin
WC: Waist circumference
β-CTX: β-C-terminal telopeptide of type I collagen
